# A prospective controlled randomized multicenter study to evaluate the severity of compensatory sweating after one-stage bilateral thoracic sympathectomy versus unilateral thoracic sympathectomy of the dominant side

**DOI:** 10.1016/j.clinsp.2026.101219

**Published:** 2026-11-05

**Authors:** Miguel Lia Tedde, Niura Noro Hamilton, Nelson Wolosker, Marina Borri Wolosker, Wolfgang W. Schmidt Aguiar, Hylas Paiva da Costa Ferreira, Fernando Luiz Westphal, Alexandre M. Rodrigues Lima, Humberto A. de Oliveira, Sergio Tadeu L.F. Pereira, Fabio de Oliviera Riuto, Guilherme C. Resende, Marina Maria Krum Brenner, Daniel de Oliveira Bonomi, Caroline E. Brero Valero, Paulo M. Pego-Fernandes

**Affiliations:** a Instituto do Coração (InCor), Hospital das Clínicas, Universidade de São Paulo, São Paulo, SP, Brazil; b Hospital das Clínicas, Universidade de São Paulo, São Paulo, SP, Brazil; c Hospital Universitário Oswaldo Cruz, Recife, PE, Brazil; d Hospital Liga Norte Riograndense Contra o Câncer, Natal, RN, Brazil; e Hospital da Universidade Federal do Amazonas, Manaus, AM, Brazil; f Hospital Geral Dr. Cesar Cals, Fortaleza, CE, Brazil; g Hospital de Base ‒ SMHS, Brasília, DF, Brazil; h Hospital Santa Isabel, Salvador, BA, Brazil; i Hospital da Universidade Federal de Grande Dourados, Dourados, MS, Brasil; j Hospital da Universidade de Brasília, Brasília, DF, Brasil; k Hospital Universitário de Santa Maria, Santa Maria, RS, Brasil; l Hospital das Clínicas da Universidade Federal de Minas Gerais, Belo Horizonte, MG, Brazil; m Empresa Brasileira de Serviços Hospitalares (SCS), Brasília, DF, Brazil

**Keywords:** Hyperhidrosis, Sympathectomy, Compensatory sweating, Quality of life, Randomized controlled trial

## Abstract

**Objective:**

To compare, in a Brazilian population, the clinical efficacy, Quality-of-Life (QoL) impact, and severity of compensatory sweating (CS; new excessive sweating in previously unaffected body regions) after one-stage Bilateral Thoracic Sympathectomy (BTS) versus Unilateral Sympathectomy on the dominant side (UniS), with additional assessment of patients who later underwent contralateral surgery (two-stage bilateral Sympathectomy, 2stS).

**Methods:**

This was a prospective, randomized, controlled, multicenter trial conducted across 11 Brazilian centers and included 163 adults with primary palmar hyperhidrosis. Participants were randomized 1:1 to BTS or UniS. From 6-months onward, UniS patients could elect contralateral sympathectomy. Sweating severity was assessed using the Hyperhidrosis Disease Severity Scale (HDSS) across predefined anatomical sites, and QoL was measured with the HidroQOL and Horn questionnaires. CS was defined as new postoperative sweating in previously unaffected areas (preoperative HDSS = 1) and graded by the magnitude of HDSS increase.

**Results:**

Baseline characteristics were generally comparable between groups. Improvement in the operated dominant hand was similar after BTS and UniS, whereas control of the non-operated hand favored BTS. In the UniS group, spontaneous contralateral improvement occurred in approximately 1 in 7 untreated hands. The proportion of patients without CS was similar in both groups (approximately 25%), but severe CS was more frequent after BTS than after UniS (40.4%vs. 21.0%; nominal, unadjusted p = 0.0344), an absolute difference of 19.4 percentage points. QoL improved in both groups, with larger and more sustained reductions in Horn and HidroQOL scores after BTS (p < 0.001). Thirty-two UniS patients underwent contralateral completion (40.0% of the randomized UniS group); post-completion outcome data were available for 18- at 8-months and 16- at 12-months. In this selected cohort, contralateral surgery produced additional HDSS and QoL improvement. CS was reassessed in 16 patients and occurred in 9 (56.2%; exact 95% CI 29.9%‒80.2%), with eight of nine cases classified as mild.

**Conclusion:**

BTS provides more complete bilateral symptom control and greater QoL improvement, but at the cost of more severe CS. UniS offers excellent control on the treated side, reduces the risk of severe CS, and supports a staged strategy in which many patients avoid a second procedure; when needed, contralateral completion can restore additional clinical and QoL gains.

## Highlights

• Bilateral surgery had better bilateral control for palmar hyperhidrosis.
• Severe compensatory hyperhidrosis was more common after bilateral sympathectomy than unilateral sympathectomy.
• Unilateral sympathectomy achieved better dominant-hand control with lower severe compensatory seweating.
• Quality-of-life improved significantly after both surgical strategies.
• A staged approach allowed some patients to avoid contralateral surgery.

## Introduction

Hyperhidrosis (HH) is a common clinical condition that affects approximately 2.8% of the general population.^1^ It is characterized by excessive and uncontrolled sweating localized to specific body regions, beyond what is physiologically necessary for thermoregulation.^2^ Among its presentations, Palmar Hyperhidrosis (PH) is particularly important because it interferes with emotional well-being, social interaction, professional activity, and manual tasks.^3^^,^^4^ PH is frequently associated with plantar and axillary hyperhidrosis, forming a broader spectrum of hyperhidrotic disease.^5^ Although benign in terms of survival, its psychosocial burden can be substantial.^6^

Bilateral video-assisted Thoracic Sympathectomy (BTS), with both sides treated during the same anesthetic session, is a widely accepted and effective surgical option for PH.^7^^,^^8^ It produces rapid symptom relief and substantial QoL improvement.^9^ However, Compensatory Sweating (CS) remains the most common and clinically relevant adverse event after sympathectomy.^10^, ^11^, ^12^, ^13^, ^14^ CS most often affects the trunk, abdomen, and back and may be influenced by the level and extent of sympathetic interruption, baseline sweating distribution, body mass index, and environmental conditions.^15^^,^^16^

To reduce CS, several authors have evaluated Unilateral Sympathectomy (UniS) or staged bilateral surgery (2stS), in which the dominant side is treated first, and the contralateral side is treated only if residual symptoms remain unacceptable. Previous studies reported high efficacy on the treated side, variable spontaneous contralateral improvement, and lower CS rates with unilateral or staged approaches, but most were retrospective or conducted in single populations.^17^, ^18^, ^19^, ^20^, ^21^, ^22^, ^23^ The only recent prospective comparison was performed in a Japanese population and reported higher satisfaction and lower CS after UniS than after BTS.^23^

Therefore, stronger evidence from other populations is needed. We conducted a randomized multicenter clinical trial across 11 Brazilian institutions to test the hypothesis that dominant-side UniS would improve palmar HH and reduce severe CS compared with BTS.

The primary aim was to compare hand sweating control and CS severity after BTS versus UniS. We also evaluated the proportion of UniS patients who chose contralateral completion, the outcomes of the 2stS subgroup, and postoperative QoL across surgical strategies.

## Materials and methods

### Study design

This was a prospective, randomized, controlled, multicenter clinical trial that enrolled 163 adult participants (age ≥ 18-years) (Fig. 1) with primary palmar HH and Body Mass Index (BMI ≤ 28 kg/m^2^). The BMI threshold was a pragmatic, prespecified decision reached by consensus among investigators before recruitment because most participating centers already used this criterion in routine clinical selection and because higher BMI has been associated with increased risk and severity of CS after sympathectomy; it was not derived from the present data. A total of 11 centers throughout Brazil participated in the study.^24^

**Fig. 1:**
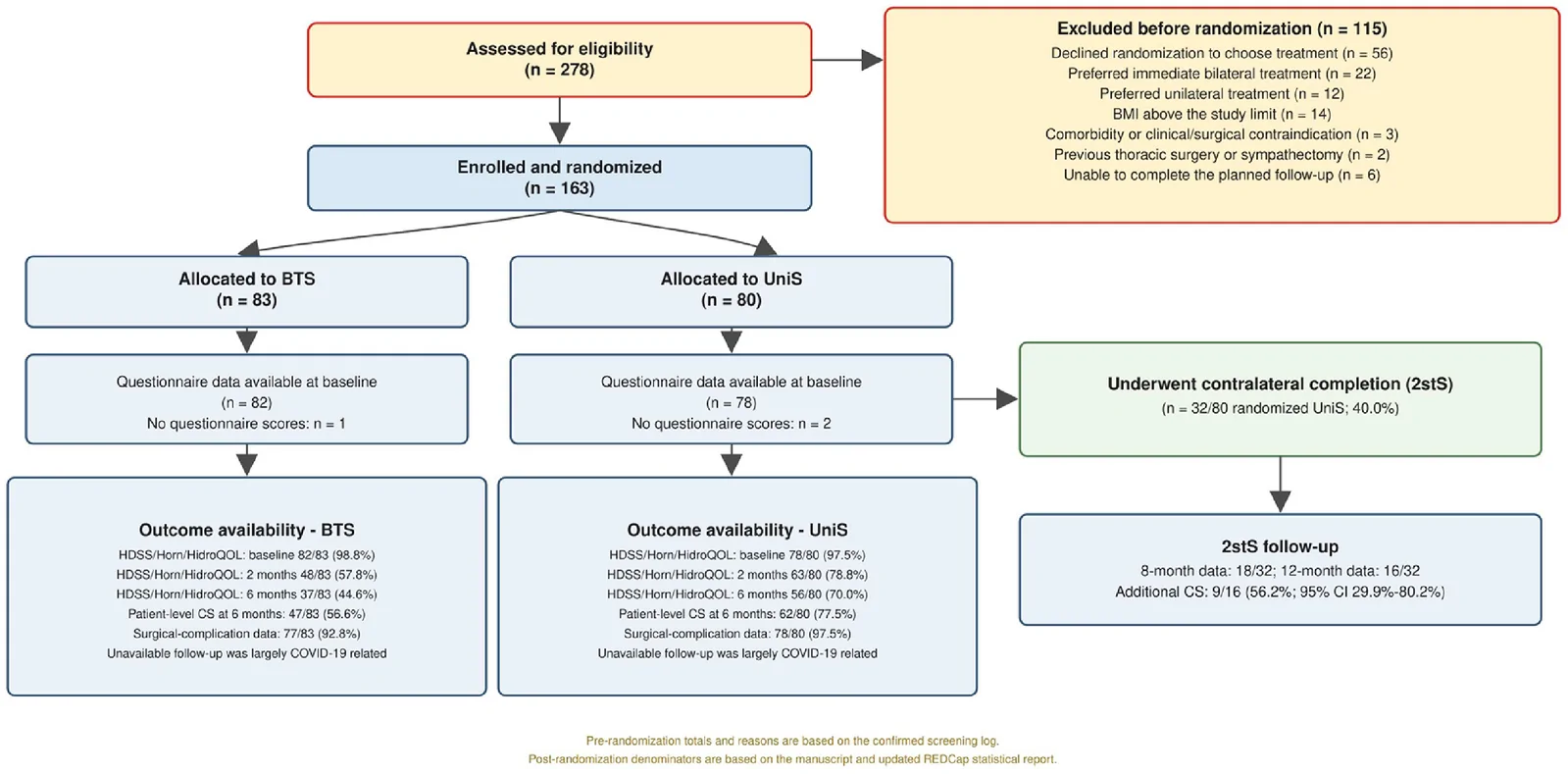
CONSORT-style flow diagram of randomization outcome availability, and staged contralateral completion.

All patients were treated according to the same protocol and in accordance with the Declaration of Helsinki. The study was approved by the relevant ethics committees and registered in Plataforma Brasil (CAAE 00,273,818.4.1001.0068) and ClinicalTrials.gov (NCT03921320). Written informed consent was obtained from every patient. The outcomes reported here are consistent with the registered trial objectives; no unplanned change in the primary clinical objectives was introduced for the present report.

Participants were randomly assigned in a 1:1 ratio to one of two treatment arms using a computer-generated random allocation sequence: BTS, defined as one-stage bilateral thoracic sympathectomy, or UniS, defined as unilateral thoracic sympathectomy on the dominant side. All eligible participants were right-handed; therefore, the right side was always treated in the UniS group. Hand dominance was established from reported habitual hand use at enrollment, without a standardized hand-dominance inventory. Patients in the UniS group who remained dissatisfied after follow-up could subsequently undergo contralateral sympathectomy and were analyzed descriptively as the 2stS subgroup.

All operations were performed under general anesthesia. The sympathectomy level was standardized at T4 because prospective comparative and randomized objective studies from our group showed palmar control comparable to T3, with less frequent or less severe CS and less excessive hand dryness after T4; these findings are consistent with subsequent meta-analytic evidence.^25^, ^26^, ^27^ The thermoablation segment was delineated between the upper edge of the fourth rib and the lower edge of the fifth costal arch, corresponding to the fourth sympathetic ganglion. In the BTS group, the same procedure was performed on the contralateral sympathetic chain during the same anesthetic session. From the sixth postoperative month onward, UniS patients who considered the result unsatisfactory could undergo contralateral surgery.

Patients in the UniS and BTS groups underwent clinical assessment, sweating intensity measurement, and QoL assessment at baseline and at 2- and 6-months after the operation. Patients who underwent a second-stage contralateral operation had two additional assessments at 2- and 6-months after the second procedure, corresponding to 8- and 12-months after the initial operation.

The primary endpoints were change in sweating severity, measured by the Hyperhidrosis Disease Severity Scale (HDSS), and change in QoL, measured by the HidroQOL and Horn questionnaires.^28^, ^29^, ^30^ Secondary endpoints included the incidence and severity of CS, as well as the proportion of UniS patients who chose contralateral completion.

To assess sweating intensity, we used the HDSS across 18 predefined body regions: right face, left face, right chest, left chest, right abdomen, left abdomen, right back, left back, right axilla, left axilla, right hand, left hand, right inguinal region, left inguinal region, right foot, and left foot. The HDSS is a validated tool for measuring HH severity and has been translated and validated for use in Portuguese.^28^ A score of 1 indicates absence of excessive sweating, a score of 2 indicates moderate HH, and scores of 3 and 4 indicate severe HH.

Palmar HH severity was defined by the HDSS score for each hand, and surgical efficacy was assessed by the change from preoperative to postoperative scores. A successful hand outcome was defined as HDSS ≤ 2 at follow-up.

CS was defined as excessive sweating that developed after sympathectomy in body regions that did not previously exhibit clinically relevant sweating. For the CS analysis, each region that was non-sweating preoperatively (HDSS = 1) was followed over time. A region that remained HDSS = 1 was classified as no CS, progression to HDSS = 2 was classified as mild CS, and progression to HDSS = 3 or 4 was classified as severe CS. This classification was applied at the regional level; for the patient-level analysis, the most severe CS grade observed in any evaluated site was used.

To measure QoL, we used two instruments developed for patients with HH: the HidroQOL and the questionnaire described by Horn.^29^^,^^30^ The HidroQOL includes 18 questions divided into activities of daily living and psychosocial domains, with total scores ranging from 0 to 36; higher scores indicate greater QoL impairment. The Horn questionnaire includes 10 closed questions addressing embarrassment, daily life, work, social events, public speaking, self-esteem, leisure, hygiene, and movement limitations; scores range from 0 to 30, with higher scores indicating greater QoL impairment.

### Data analysis

Descriptive analyses were performed for all variables. Normally distributed continuous data were presented as mean ± Standard Deviation (SD), and non-normally distributed data as median and interquartile range (IQR 25th‒75th percentiles). Normality was assessed using the Shapiro-Wilk test and visual inspection of histograms. Categorical variables were summarized as counts and percentages.

The primary randomized comparison followed a modified intention-to-treat principle: all randomized patients who underwent the assigned initial surgical strategy and had baseline data were retained in their assigned group. Outcome-specific analyses used available cases, and denominators are provided in each table because the number of patients with complete follow-up varied by outcome and time point, largely because part of the follow-up occurred during the COVID-19 pandemic. No imputation was performed for missing follow-up outcomes.

Between-group comparisons of continuous variables used the unpaired Student *t*-test or Mann-Whitney *U* test, as appropriate. Within-group comparisons used paired tests or repeated-measures models. Categorical variables were compared using Chi-Square tests or Fisher’s exact tests when expected cell counts were small. Mixed models, including group, time point, and group-by-time interaction, were used for repeated outcome comparisons when appropriate. Non-randomized comparisons involving the 2stS subgroup were interpreted as descriptive/exploratory because the decision to undergo contralateral surgery was based on patient preference.

Prespecified main randomized comparisons were evaluated using a two-sided alpha of 0.05 without a formal multiplicity adjustment or hierarchical testing strategy; the corresponding p-values are therefore nominal. We interpreted multiple secondary comparisons across time points and anatomical regions as exploratory, emphasizing effect size, direction, and clinical relevance rather than p-values alone. Visit-specific descriptive summaries and the 6-month patient-level CS comparison used available observations, and no missing outcomes were imputed. Longitudinal analyses incorporated all observed repeated measurements, so participants were not excluded solely because a follow-up visit was missing. When estimable, models included fixed effects for group, time, and the group-by-time interaction, plus a patient-level random effect. Horn and HidroQOL scores were modeled using negative-binomial distributions and the global CS score using a gamma distribution. Because of sparse categories, HDSS was grouped as 1‒2 versus 3‒4 and analyzed using bias-reduced binary logistic regression. Updated analyses were performed in R version 4.2.2, including the brglm package for the bias-reduced models.

## Results

A total of 163 patients were analyzed, and their baseline characteristics are summarized in Table 1. Most patients were women (62%) in the third decade of life (median age, 24.1-years). The Heart Institute contributed 26.4% of the sample, the University Hospital Oswaldo Cruz realized 11.5%, and the remaining participants were distributed across the other 9 participating centers, contributing 0.6% to 3.9% each. There was a broad distribution of HH phenotypes, with palmar involvement present in all cases. The median BMI was 24.0 kg/m^2^, with no significant difference between groups. Most patients were Caucasian (73.1%), disease onset occurred in childhood in 83.5% of patients, and the median disease duration was 16-years.

**Table 1:** Baseline patient characteristics according to surgical group.

**Variable**	**Total (n** **=** **163)**	**BTS (n** **=** **83)**	**UniS (n** **=** **80)**	**p-value**
Age (years)	24.1 [20.9; 30.5]	23.3 [21.1; 28.5]	26.0 [20.9; 31.7]	0.179(a)
Gender				0.106
Male	62 (38.0%)	37 (44.6%)	25 (31.2%)	
Female	101 (62.0%)	46 (55.4%)	55 (68.8%)	
Type of hyperhidrosis				0.037
Palmar	24 (14.7%)	10 (12.0%)	14 (17.5%)	
Palmar + Axillary	7 (4.3%)	7 (8.4%)	0 (0.0%)	
Palmar + Plantar	91 (55.8%)	48 (57.8%)	43 (53.8%)	
Palmar + Axillary + Plantar	41 (25.2%)	18 (21.7%)	23 (28.8%)	
BMI	24.0 [21.0; 26.0]	23.0 [20.5; 26.0]	24.0 [21.0; 25.2]	>0.999
Ethnicity				0.725
Caucasian	117 (73.1%)	61 (74.4%)	56 (71.8%)	
African descent	43 (26.9%)	21 (25.6%)	22 (28.2%)	
Onset of illness				
Childhood	132 (83.5%)	69 (84.1%)	63 (82.9%)	
Adolescence	24 (15.2%)	12 (14.6%)	12 (15.8%)	
Adult	2 (1.3%)	1 (1.2%)	1 (1.3%)	
Duration of illness (years)	16.0 [12.2; 22.0]	15.0 [11.0; 21.0]	20.0 [13.5; 25.0]	0.157(a)
HDSS dominant hand before surgery				0.056
Grade 1	1 (0.6%)	1 (1.2%)	0 (0.0%)	
Grade 2	0 (0.0%)	0 (0.0%)	0 (0.0%)	
Grade 3	16 (10.0%)	4 (4.9%)	12 (15.4%)	
Grade 4	143 (89.4%)	77 (93.9%)	66 (84.6%)	
HORN score before surgery	20.3 ± 5.6	19.1 ± 6.5	19.7 ± 6.1	0.210
HidroQOL before surgery	27.0 [23.0; 31.0]	28.0 [23.0; 31.0]	27.0 [21.2; 30.0]	0.386(a)

Values are presented as n (%), mean ± SD, or median [IQR]. Fisher’s exact or Chi-Square tests were used for categorical variables; Mann-Whitney *U* tests were used for non-normally distributed continuous variables when indicated.

Preoperatively, 89.4% of patients had HDSS grade 4 in the dominant hand (93.9% in BTS and 84.6% in UniS; p = 0.056). Baseline QoL was similarly impaired in both groups: mean Horn score was 20.3 ± 5.6 (p = 0.210), and median HidroQOL was 27.0 (IQR 23.0‒31.0; p = 0.386). The distribution of HH type differed statistically between groups (p = 0.037), mainly because palmar-plus-axillary disease was more frequent in the BTS group; this imbalance was considered in the interpretation because axillary symptoms may influence the decision to request second-stage surgery.

Table 2 and Fig. 2 summarize the proportion of patients achieving HDSS 1‒2 in each hand at baseline, 2-months, and 6-months. Before surgery, nearly all patients in both groups Had Severe Disease (HDSS 3‒4) in both hands. At 2-months, more than 93% of BTS patients improved to HDSS 1‒2 in both hands, whereas in the UniS group, 92.1% reached HDSS 1‒2 in the operated hand and 20.6% in the contralateral hand. At 6-months, 91.9% of BTS patients remained at HDSS 1‒2 bilaterally; in the UniS group, 91.1% were HDSS 1‒2 in the operated hand and 14.3% in the untreated hand. Thus, treated limbs showed marked improvement, whereas 8 of 56 untreated hands improved from baseline HDSS 3‒4 to the prespecified successful-hand threshold of HDSS 1‒2 (14.3%; exact 95% CI 6.4%‒26.2%).

**Table 2:** Evolution of HDSS hand scores in patients undergoing BTS versus UniS.

**Time point**	**HDSS category**	**BTS right hand**	**BTS left hand**	**UniS sympathectomized hand**	**UniS non-sympathectomized hand**
Before	1 or 2	1 (1.2%)	1 (1.2%)	0 (0.0%)	0 (0.0%)
Before	3 or 4	81 (98.8%)	81 (98.8%)	78 (100.0%)	78 (100.0%)
After 2-months	1 or 2	46 (95.8%)	45 (93.8%)	58 (92.1%)	13 (20.6%)
After 2-months	3 or 4	2 (4.2%)	3 (6.2%)	5 (7.9%)	50 (79.4%)
After 6-months	1 or 2	34 (91.9%)	34 (91.9%)	51 (91.1%)	8 (14.3%)
After 6-months	3 or 4	3 (8.1%)	3 (8.1%)	5 (8.9%)	48 (85.7%)

**Fig. 2:**
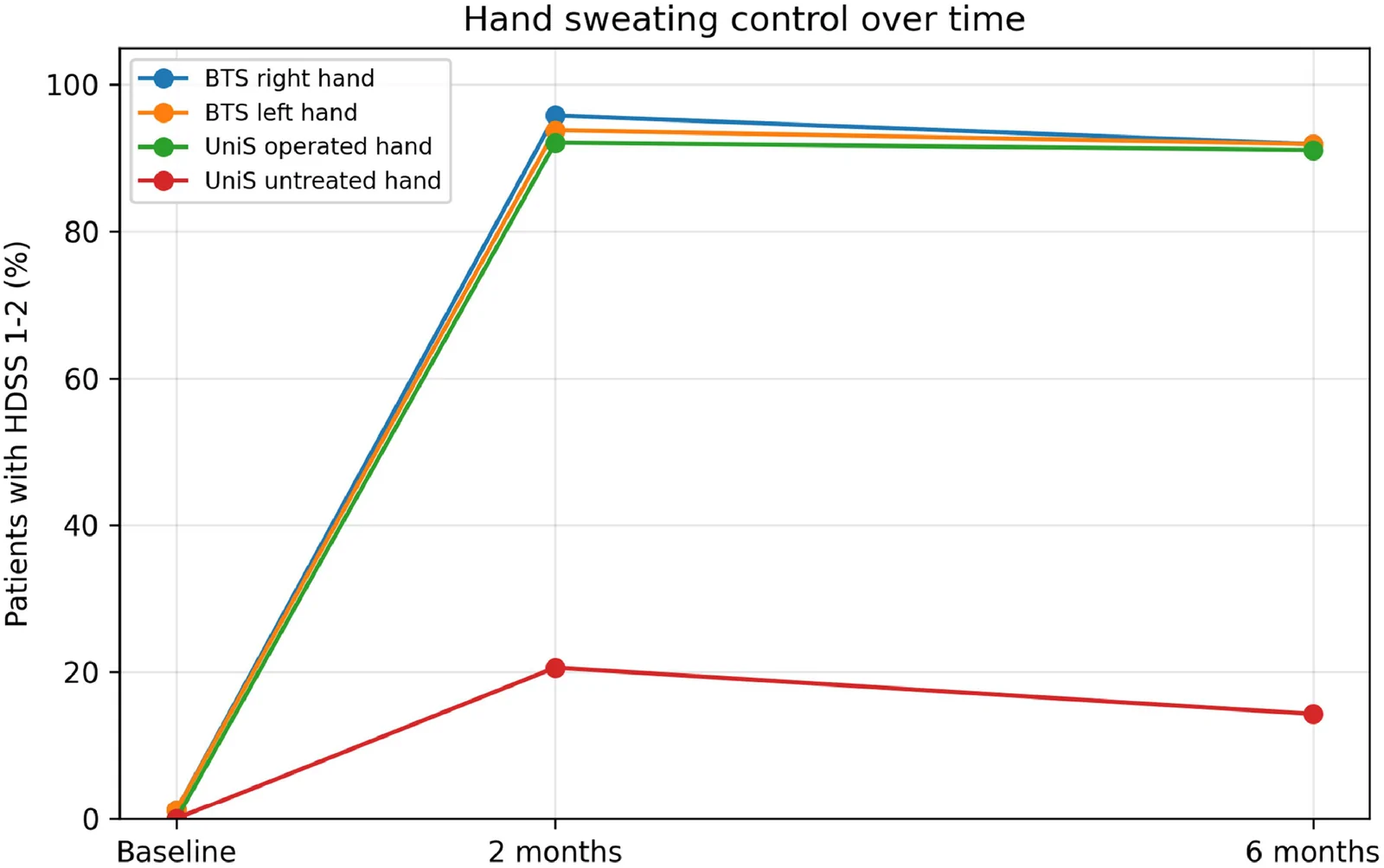
Proportion of patients with successful hand outcome (HDSS ≤2) at baseline, 2-months, and 6-months.

Table 3 compares postoperative HDSS scores between groups. Dominant-hand scores were similar between BTS and UniS at 2- and 6-months (median = 1.0; p > 0.4), indicating comparable control on the operated side. In contrast, the non-dominant hand favored BTS at both follow-up points (median 1.0 after BTS vs. 4.0 at 2-months and 3.0 at 6-months after UniS; p < 0.001).

**Table 3:** Hand sweating severity (HDSS) before surgery and at 2- and 6-months after BTS versus UniS.

Variable	BTS (n = 82)	UniS (n = 78)	p-value
HDSS right hand before	4.0 [4.0; 4.0]	4.0 [4.0; 4.0]	
HDSS right hand after 2-months	1.0 [1.0; 1.0]	1.0 [1.0; 2.0]	0.482
HDSS right hand after 6-months	1.0 [1.0; 1.0]	1.0 [1.0; 2.0]	0.944
HDSS left hand before	4.0 [4.0; 4.0]	4.0 [4.0; 4.0]	
HDSS left hand after 2-months	1.0 [1.0; 1.0]	4.0 [3.0; 4.0]	<0.001
HDSS left hand after 6-months	1.0 [1.0; 1.0]	3.0 [3.0; 4.0]	<0.001

Values are presented as median [IQR]. Between-group comparisons were obtained from mixed models including group, time point, and group-by-time interaction when appropriate.

Table 4 shows within-group changes in hand HDSS scores from baseline. In both groups, the operated/right hand showed a consistent median reduction of approximately 3-points at 2- and 6-months (p < 0.001). In the BTS group, the left hand also improved by approximately 3-points at both follow-up points. In the UniS group, the non-operated hand showed no clinically meaningful improvement at 6-months, confirming that UniS provides sustained benefit mainly in the treated hand.

**Table 4:** Change from baseline in HDSS hand scores within each group.

**Variable**	**Moment**	**BTS median [IQR] or mean (95% CI)**	**BTS p-value**	**UniS median [IQR] or mean (95% CI)**	**UniS p-value**
HDSS right hand	Post 2-months ‒ Pre	−3.0 [−3.0; −2.0]	<0.001	−3.0 [−3.0; −2.0]	<0.001
HDSS right hand	Post 6-months ‒ Pre	−3.0 [−3.0; −2.0]	<0.001	−3.0 [−3.0; −2.0]	<0.001
HDSS left hand	Post 2-months ‒ Pre	−3.0 [−3.0; −2.0]	<0.001	0.0 [−1.0; 0.0]	0.028
HDSS left hand	Post 6-months ‒ Pre	−3.0 [−3.0; −2.0]	<0.001	0.0 [−1.0; 0.0]	0.064

Values are presented as median [IQR] or mean (95% CI).

The 14.3% untreated-hand response should not be interpreted as proof of a physiologic cross-side effect. Possible explanations include central autonomic adaptation, behavioral adaptation, environmental or seasonal variation, and measurement variability. Nevertheless, the change from HDSS 3‒4 to HDSS 1‒2 met the prespecified patient-reported threshold for clinical success; objective sweat measurement was not available to distinguish among these mechanisms.

Table 5 and Fig. 3 compare patient-level CS severity at 6-months. The proportion of patients without CS was virtually identical in both groups (25.5% after BTS and 25.8% after UniS). However, mild CS was more frequent after UniS (53.2%vs. 34.1%), whereas severe CS was more frequent after BTS (40.4%vs. 21.0%; nominal, unadjusted p = 0.0344). The absolute difference in severe CS was 19.4 percentage points (approximate 95% CI 2.2‒36.8 percentage points), which is clinically relevant because severe CS may reduce the perceived benefit despite adequate palmar control.

**Table 5:** Distribution of compensatory sweating grades 6-months after surgery.

**Compensatory sweating**	**BTS (n** **=** **47)**	**UniS (n** **=** **62)**	**p-value (Fisher test)**
Absent	12 (25.5%)	16 (25.8%)	
Mild	16 (34.1%)	33 (53.2%)	
Severe	19 (40.4%)	13 (21.0%)	0.0344

**Fig. 3:**
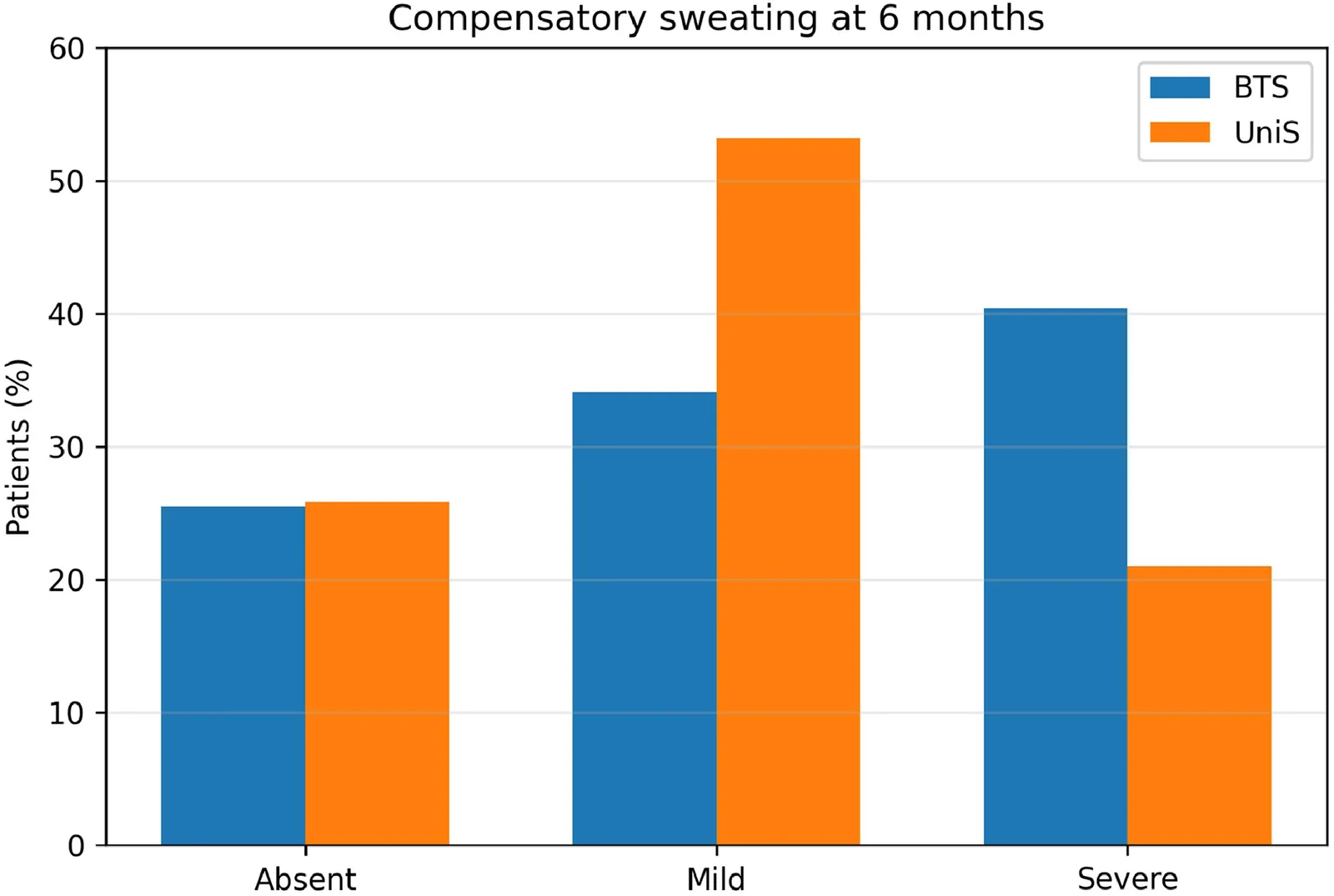
Distribution of patient-level compensatory sweating severity at 6-months after BTS versus UniS.

Visit-level questionnaire outcomes (hand HDSS, Horn, and HidroQOL) were available at baseline for 160 of 163 randomized participants (98.2%; 82/83 BTS and 78/80 UniS), at 2-months for 111/163 (68.1%; 48/83 and 63/80), and at 6-months for 93/163 (57.1%; 37/83 and 56/80). Patient-level CS outcomes at 6-months were available for 109/163 (66.9%; 47/83 and 62/80, respectively) (Table 6).

**Table 6:** Outcome-specific data availability after randomization.

**Outcome/time point**	**BTS, n/n (%)**	**UniS, n/n (%)**	**Total, n/n (%)**
Hand HDSS, baseline	82/83 (98.8)	78/80 (97.5)	160/163 (98.2)
Hand HDSS, 2-months	48/83 (57.8)	63/80 (78.8)	111/163 (68.1)
Hand HDSS, 6-months	37/83 (44.6)	56/80 (70.0)	93/163 (57.1)
Patient-level CS, 6-months	47/83 (56.6)	62/80 (77.5)	109/163 (66.9)
Horn, baseline	82/83 (98.8)	78/80 (97.5)	160/163 (98.2)
Horn, 2-months	48/83 (57.8)	63/80 (78.8)	111/163 (68.1)
Horn, 6-months	37/83 (44.6)	56/80 (70.0)	93/163 (57.1)
HidroQOL, baseline	82/83 (98.8)	78/80 (97.5)	160/163 (98.2)
HidroQOL, 2-months	48/83 (57.8)	63/80 (78.8)	111/163 (68.1)
HidroQOL, 6-months	37/83 (44.6)	56/80 (70.0)	93/163 (57.1)

Percentages use the full randomized denominator. The updated statistical report indicates common visit-level availability for hand HDSS, Horn, and HidroQOL. Patient-level CS was available for a different set of participants. Unavailability was largely related to interrupted pandemic-era follow-up.

An extreme-case sensitivity analysis classified all missing BTS outcomes as non-severe and all missing UniS outcomes as severe, yielding severe-CS rates of 22.9% versus 38.8%. Under the opposite assumptions, rates were 66.3% versus 16.3%. Because the direction can reverse under extreme assumptions, the observed available-case difference should be interpreted cautiously.

At the regional level, new postoperative sweating in areas that were normal preoperatively occurred mainly in the trunk and adjacent regions (Supplementary Table 1). The highest overall rates of new sweating were in the abdominal and hemidorsal regions after BTS and in the hemidorsal and thoracic regions after UniS. Among regions with preoperative HDSS = 1, most areas remained stable, but progression to HDSS 3‒4 occurred in 10.8% of bilateral observations and 7.7% of unilateral observations.

Table 7 and Fig. 4 compare Horn and HidroQOL scores between groups. Preoperatively, scores were comparable. At 2-months, the BTS group had markedly lower median Horn (1.5 vs. 8.0) and HidroQOL (6.0 vs. 18.0) scores than the UniS group (p < 0.001), indicating a stronger early QoL response. This difference persisted at 6-months, with lower Horn (5.0 vs. 12.0) and HidroQOL (8.0 vs. 17.0) scores after BTS (p < 0.001). Table 8 confirms that both groups improved from baseline, but the magnitude of QoL improvement was greater after BTS.

**Table 7:** Postoperative Horn and HidroQOL scores according to surgical group.

**Variable**	**BTS (n** **=** **82)**	**UniS (n** **=** **78)**	**p-value**
Horn score before	21.0 [16.0; 25.0]	20.0 [15.0; 24.0]	
Horn score after 2-months	1.5 [0.0; 5.0]	8.0 [4.0; 15.5]	<0.001
Horn score after 6-months	5.0 [1.0; 9.0]	12.0 [4.0; 16.0]	<0.001
HidroQOL before	28.0 [23.0; 31.0]	27.0 [21.2; 30.0]	
HidroQOL after 2-months	6.0 [3.0; 15.0]	18.0 [11.5; 23.0]	<0.001
HidroQOL after 6-months	8.0 [5.0; 14.0]	17.0 [10.8; 25.0]	<0.001

Values are presented as median [IQR].

**Fig. 4:**
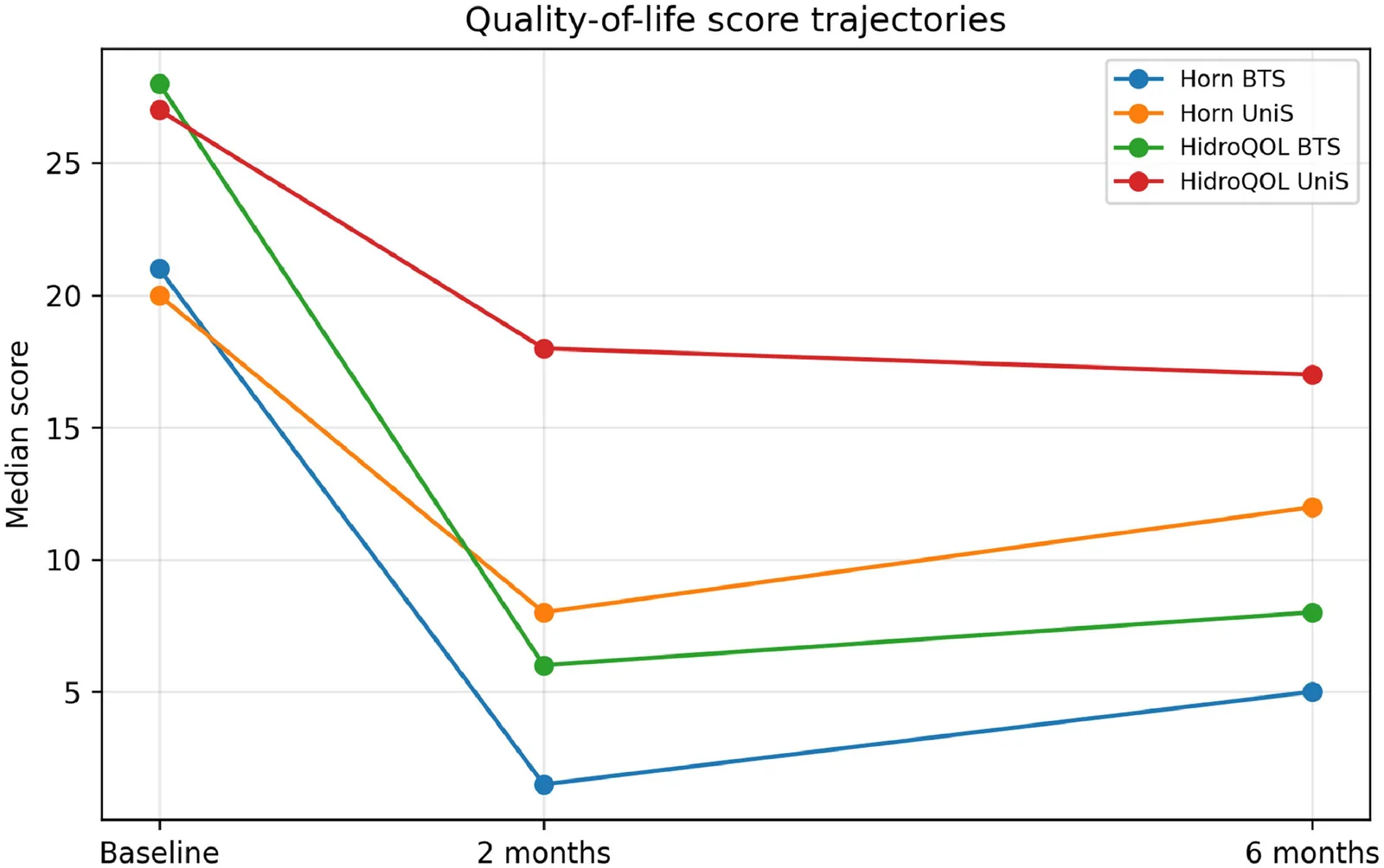
Median Horn and HidroQOL scores over time according to randomized surgical group.

**Table 8:** Change from baseline in Horn and HidroQOL scores within each group.

**Variable**	**Moment**	**BTS median [IQR] or mean (95% CI)**	**BTS p-value**	**UniS median [IQR] or mean (95% CI)**	**UniS p-value**
Horn score	Post 2-months ‒ Pre	−23.5 [−27.0; −17.8]	<0.001	−14.0 [−21.0; −11.0]	<0.001
Horn score	Post 6-months ‒ Pre	−19.0 [−22.0; −17.0]	<0.001	−15.0 [−18.0; −11.0]	<0.001
HidroQOL	Post 2-months ‒ Pre	−17.0 [−23.0; −11.8]	<0.001	−7.0 [−13.0; −2.0]	<0.001
HidroQOL	Post 6-months ‒ Pre	−16.0 [−21.0; −10.0]	<0.001	−6.5 [−15.0; −2.8]	<0.001

Values are presented as median [IQR] or mean (95% CI).

After the initial procedure, 32 of 80 randomized UniS patients underwent contralateral surgery (40.0%). Post-completion outcome data were available for 18- at 8-months and 16- at 12-months. Among the 18 patients assessed at 8-months, all showed improvement in sweating severity after contralateral completion. Among those with preoperative HDSS 4 in the contralateral hand (n = 13), postoperative scores improved to grade 1 in 8 patients, grade 2 in 2 patients, and grade 3 in 1 patient. Among patients with preoperative HDSS 3 (n = 4), three improved to grade 1 and one to grade 2. One patient with preoperative HDSS 2 improved to grade 1 but requested the second procedure primarily because of severe contralateral axillary symptoms (HDSS 4). CS after the second procedure occurred in 9 of 16 patients with available CS reassessment (56.2%; exact 95% CI 29.9%‒80.2%): seven had mild CS at a single site, one had mild CS at two sites, and one developed intense CS involving two evaluated sites. Because post-completion outcomes derive from small, incomplete, self-selected follow-up samples, these findings are exploratory and hypothesis-generating.

Table 9 tracks HDSS, Horn, and HidroQOL trajectories in the 2stS subgroup. The dominant hand, which was operated first, showed early and sustained HDSS reduction. The contralateral hand did not significantly improve at 2- or 6-months but improved sharply after the second procedure, with a median 3-point decrease at 8- and 12-months (p < 0.001). Horn and HidroQOL scores followed the same pattern: modest improvement after the first operation and marked additional improvement after contralateral completion.

**Table 9:** HDSS, Horn, and HidroQOL trajectories in patients who underwent staged bilateral Sympathectomy (2stS).

**Variable**	**2stS median [IQR]**	**Change from baseline**	**p-value**
HDSS right hand before	4.0 [4.0; 4.0]		
HDSS right hand after 2-months	1.0 [1.0; 2.0]	−3.0 [−3.0; −2.0]	<0.001
HDSS right hand after 6-months	1.0 [1.0; 2.0]	−2.5 [−3.0; −2.0]	<0.001
HDSS right hand after 8-months	1.0 [1.0; 1.0]	−3.0 [−3.0; −2.0]	<0.001
HDSS right hand after 12-months	1.0 [1.0; 1.2]	−3.0 [−3.0; −2.0]	<0.001
HDSS left hand before	4.0 [4.0; 4.0]		
HDSS left hand after 2-months	4.0 [3.0; 4.0]	0.0 [−1.0; 0.0]	0.083
HDSS left hand after 6-months	4.0 [3.0; 4.0]	0.0 [−1.0; 0.0]	0.175
HDSS left hand after 8-months	1.0 [1.0; 1.0]	−3.0 [−3.0; −2.0]	<0.001
HDSS left hand after 12-months	1.0 [1.0; 1.2]	−3.0 [−3.0; −2.0]	<0.001
Horn score before	21.5 [16.0; 26.2]		
Horn score after 2-months	11.5 [4.2; 19.5]	−13.0 [−18.0; −9.0]	<0.001
Horn score after 6-months	13.5 [10.0; 16.8]	−14.0 [−17.0; −11.0]	<0.001
Horn score after 8-months	1.0 [0.0; 2.8]	−25.0 [−27.0; −22.5]	<0.001
Horn score after 12-months	2.5 [0.0; 9.0]	−24.0 [−25.2; −21.2]	<0.001
HidroQOL before	27.5 [24.8; 32.0]		
HidroQOL after 2-months	19.0 [14.5; 27.0]	−6.5 [−11.0; −2.0]	0.002
HidroQOL after 6-months	22.0 [15.2; 26.0]	−5.5 [−10.5; −3.0]	0.004
HidroQOL after 8-months	5.0 [2.0; 10.8]	−19.5 [−25.0; −17.0]	<0.001
HidroQOL after 12-months	5.0 [1.8; 18.2]	−21.0 [−24.2; −11.8]	<0.001

The 2stS subgroup included 18 patients; denominators may vary by time point and outcome because available-case analyses were used. Values are presented as median [IQR].

Among 155 patients with surgical-complication records (77 BTS and 78 UniS), postoperative pneumothorax occurred in 7 (4.5%; 5/77 vs. 2/78; p = 0.276), pleural effusion in 3 (1.9%; 2/77 vs. 1/78; p = 0.620), and another postoperative complication in 1 (0.6%; 1/77 vs. 0/78; p = 0.497). Among 10 patients with a completed drainage-after-complication field, 8 were drained for a mean of 1.9 ± 0.8 days. Recorded intraoperative events included right-sided laceration in 5/155 (3.2%; 4/77 vs. 1/78) and right-sided bleeding in 2/155 (1.3%; 2/77 vs. 0/78); left-sided laceration, bleeding, and another event were each recorded in one BTS patient. Limb paresis occurred in 6/138 (4.3%; 2/65 vs. 4/73; p = 0.684) and gustatory sweating in 3/138 (2.2%; 1/65 vs. 2/73; p > 0.999). No between-group comparison for these recorded complications was statistically significant. Horner syndrome, wound infection, reoperation, readmission, and death did not happen.

## Discussion

In this Brazilian multicenter randomized trial, BTS and UniS produced similar control of sweating in the operated dominant hand, but BTS provided superior control of the non-dominant hand and greater QoL improvement. This benefit came at the cost of more severe CS: severe CS occurred in 40.4% of BTS patients and 21.0% of UniS patients at 6-months. Thirty-two of 80 randomized UniS patients underwent contralateral completion, but post-completion outcomes were available for only 18- at 8-months and 16- at 12-months. The additional clinical and QoL improvement and predominantly mild incremental CS observed after 2stS therefore derive from small, selected follow-up samples and should be considered exploratory.

Excessive palmar sweating can substantially impair daily life, leading to occupational limitations, social embarrassment, and difficulties in interpersonal relationships.^3^^,^^4^^,^^31^ First-line treatment often includes oxybutynin hydrochloride, which improves symptoms in many patients,^32^^,^^33^ but surgery remains the most effective option when medical therapy fails or is not tolerated.^7^^,^^13^ The central question is therefore not whether sympathectomy works, but how to balance palmar control against the risk of CS.^34^^,^^35^

The present design tested two clinically relevant strategies: immediate bilateral treatment versus dominant-side treatment with the possibility of later contralateral completion. This is important for patient counseling. BTS should be presented as the option with the greatest chance of immediate bilateral symptom control and larger QoL gains, but also a higher risk of severe CS. UniS should be presented as an option that reliably controls the treated hand and may reduce severe CS, but at the price of persistent sweating in the untreated hand for most patients. The staged approach gives patients time to decide whether the residual burden justifies a second procedure.

Our findings are consistent with previous reports showing high efficacy of unilateral or staged sympathectomy on the treated side and lower CS rates than with one-stage bilateral treatment.^17^, ^18^, ^19^, ^20^, ^21^, ^22^ Compared with the prospective Japanese study by Shih et al.,^23^ our study also found less severe CS after UniS; in the present trial, 40.0% of randomized UniS patients ultimately underwent contralateral completion. The overall incidence of CS in our cohort was higher. Documented differences in the sympathetic level treated (T3 in the Japanese study vs. T4 here), follow-up duration, case mix, and our systematic site-specific HDSS definition and assessment may contribute to the different estimates; the present data do not permit attribution to climate or ethnicity.

The inclusion of 11 centers across Brazil increases external validity by incorporating geographic, ethnic, and climatic variability. The Heart Institute contributed 26.4% of the sample, and no interpretation should ignore the possibility of center effects. Nevertheless, the multicenter recruitment, standardized T4 technique, and use of validated patient-reported instruments strengthen the generalizability of the randomized comparison. The applicability of trial findings depends on how well the study population and clinical settings reflect routine practice.^36^ Nationwide Brazilian data also document the use of thoracic sympathectomy in the public health system.^37^

All patients underwent T4 sympathectomy, a level supported by prospective comparative and randomized objective studies from our group and by meta-analytic evidence showing effective palmar control with a lower burden of CS than higher or more extensive interruption.^25^, ^26^, ^27^ Because both randomized groups were treated at the same level, the difference in severe CS is more likely related to unilateral versus bilateral interruption than to differences in surgical level. This supports the biological plausibility that preserving the contralateral sympathetic chain may reduce the severity of postoperative CS.

The site-specific analysis reinforces the clinical nature of CS. New sweating in previously normal regions occurred mainly in the trunk and adjacent areas, including hemidorsal, abdominal, thoracic, gluteal, inguinal, and thigh regions. These regions should be discussed explicitly before surgery, because patients often judge the operation not only by palmar dryness but also by whether new sweating affects clothing, work, or social comfort. Moreover, for analytic purposes, postoperative HDSS 2 was classified as mild CS, but it may still be clinically important when sweating affects clothing, work, or social activities.

QoL findings show the trade-off clearly. BTS produced larger reductions in Horn and HidroQOL scores at 2- and 6-months, likely because both hands were treated immediately, and bimanual activities improved more completely. UniS also improved QoL compared with baseline, but residual sweating on the untreated hand probably limited the magnitude of improvement. Therefore, patient selection should incorporate the relative importance of immediate bilateral dryness versus risk tolerance for CS.

Practical patient selection may be guided as follows. BTS may be preferable for patients with severe bilateral palmar impairment, major occupational need for both hands, low tolerance for residual hand sweating, and acceptance of higher CS risk. UniS may be preferable for patients whose main limitation is the dominant hand, those particularly concerned about CS, or those willing to accept a staged decision. The 2stS strategy is best viewed not as a separate randomized treatment arm but as a patient-centered pathway for UniS patients who remain symptomatic after the first operation. Table 10 summarizes these considerations as a non-prescriptive shared-decision framework.

**Table 10:** Practical shared-decision counseling framework.

**Counseling domain**	**One-stage BTS**	**UniS with optional contralateral completion**
Patient profile most likely to value strategy	Severe bilateral impairment; major occupational need for both hands; low tolerance of residual contralateral sweating.	Dominant-hand symptoms are the principal limitation; high concern about severe CS; accepts staged decision-making and possible second procedure.
Hand control at 6-months	HDSS ≤2 in 91.9% of each hand among participants with available data.	HDSS ≤2 in 91.1% of treated hands; spontaneous HDSS ≤2 in 14.3% of untreated hands among participants with available data.
Severe CS at 6-months	40.4% (19/47) among participants with available CS data.	21.0% (13/62) among participants with available CS data.
QoL trade-off	Larger early and 6-month improvement in Horn and HidroQOL scores; balanced against higher severe-CS risk.	Meaningful QoL improvement, generally smaller than BTS while the contralateral hand remains untreated.
Need for another operation	No planned second-side procedure.	32/80 randomized patients (40.0%) underwent contralateral completion; outcome data were available for 18 at 8-months and 16 at 12-months. Results are exploratory and selected.
Key preoperative discussion	Possibility that new trunk/adjacent-region sweating may offset some benefit despite excellent bilateral hand control.	Persistent untreated-hand sweating is expected in most patients; benefits of lower severe-CS risk must be weighed against a possible second anesthetic/procedure.

This framework summarizes observed trial outcomes and supports shared decision-making; it is not a prescriptive treatment algorithm. Percentages are available-case estimates and should be interpreted with the reported missingness.

Several limitations should be acknowledged. First, neither patients nor outcome assessors were blinded. Blinding is difficult in surgical laterality trials, but lack of blinding may influence subjective outcomes such as CS severity and QoL scores. Second, follow-up for the primary randomized comparison was limited to 6-months and may not capture late-onset or progressive CS, palmar recurrence, or changes in patient adaptation. No additional prespecified long-term randomized follow-up was available for this report; longer follow-up is warranted.^38^, ^39^, ^40^

Third, part of the study was conducted during the COVID-19 pandemic, which limited recruitment and follow-up. Pandemic-related reductions in vascular procedures, including sympathectomies for hyperhidrosis, were also documented in the Brazilian public health system.^41^^,^^42^ At 6-months, visit-level hand HDSS, Horn, and HidroQOL data were available for 93/163 randomized participants (57.1%; 37/83 BTS and 56/80 UniS), whereas patient-level CS data were available for 109/163 (66.9%; 47/83 and 62/80); outcome denominators therefore vary across analyses, and missingness was differential between groups. Visit-specific summaries and the 6-month CS comparison used available observations, while longitudinal models incorporated all observed repeated measurements. No outcomes were imputed because a defensible missing-at-random assumption could not be established. An extreme-case analysis showed that the direction of the severe-CS difference could reverse if all missing BTS outcomes were assumed non-severe and all missing UniS outcomes severe (22.9%vs. 38.8%), whereas the opposite assumptions strengthened the observed difference (66.3%vs. 16.3%). Missingness may therefore materially affect the estimate, which should be interpreted cautiously. Fourth, center effects could not be fully modeled because of the number of centers and variable recruitment per site; the largest center contributed 26.4% of patients, and the remaining patients were distributed across 10 institutions.

Fifth, the 2stS cohort was not randomized. The decision to undergo contralateral completion was based on patient preference and residual symptoms, introducing selection bias. Patients choosing 2stS may have had greater residual burden or lower tolerance for untreated-side sweating than patients who did not choose completion. Although 32 patients underwent the procedure, outcome data were available for only 18- at 8-months and 16- at 12-months; only 16 had CS reassessment, and the additional-CS estimate was imprecise (56.2%; exact 95% CI 29.9%‒80.2%). Therefore, 2stS findings should be interpreted as exploratory and hypothesis-generating rather than as a randomized comparison with BTS.

Sixth, outcomes relied on self-reported instruments rather than objective sweating measurements. Objective sweat quantification is difficult in routine clinical settings because sweating varies over time and with environment, but self-report remains subject to recall and perception bias. Patient satisfaction was not measured separately, and the observed QoL changes should not be interpreted as a satisfaction rate. Finally, some participants had associated axillary hyperhidrosis, and in at least one case the indication for second-stage surgery was driven by axillary symptoms rather than the contralateral hand. This may have influenced crossover rates and should be considered when counseling patients with combined palmar-axillary disease.

## Conclusion

BTS provided immediate and substantial control of sweating in both hands and produced the largest QoL improvement, but it was associated with a higher frequency of severe CS. UniS produced robust and durable control of the treated dominant hand, reduced severe CS, and allowed many patients to avoid a second procedure.

When used as a staged strategy, UniS followed by contralateral completion only when needed can provide additional clinical and QoL gains while preserving individualized decision-making. These findings support a shared decision-making model in which BTS, UniS, and 2stS are presented as distinct strategies with different trade-offs in symptom control, CS risk, and patient priorities.

## Generative AI statement

The authors used generative artificial intelligence only for language-editing support. AI was not used for study design, data collection, statistical analysis, data interpretation, or generation of clinical data. The authors reviewed and edited all output and take full responsibility for the content of the publication.

## Preprint disclosure

Tedde ML, Hamilton NN, Wolosker MB, Wolosker N, Aguiar WWS, Ferreira HPC, Westphal FL, Lima AMR, Oliveira HA, Pereira STLF, Riuto FO, Resende GC, Brenner MMK, Bonomi DO, Valero CEB, Fernandes PMP. A prospective controlled randomized multicenter study to evaluate severity of compensatory sweating after one-stage bilateral thoracic sympathectomy versus unilateral thoracic sympathectomy on the dominant side. medRxiv. 2026. d oi:10.64898/2026.02.18.26346562.^42^ This preprint represents an earlier, non-peer-reviewed version of the present manuscript; the submitted revised version includes corrected tables, new figures, expanded statistical reporting, and expanded limitations.

## Funding

This research did not receive any specific grant from funding agencies in the public, commercial, or not-for-profit sectors.

## Declaration of competing interest

The authors declare that they have no known competing financial interests or personal relationships that could have appeared to influence the work reported in this paper.

## Data Availability

The data underlying this article will be shared on reasonable request to the corresponding author.
