# Supplementary material for: A prospective controlled randomized multicenter study to evaluate the severity of compensatory sweating after one-stage bilateral thoracic sympathectomy versus unilateral thoracic sympathectomy of the dominant side
Source: Clinics (Sao Paulo). 2026 Nov 5;81:101219. doi: 10.1016/j.clinsp.2026.101219 (PMC13760101; doi:10.1016/j.clinsp.2026.101219)
Supplement: Supplementary file 1 [file mmc1.docx]

CLINICS-D-26-01389_Supplementary Material

**Supplementary Table 1** Regional postoperative evolution among anatomical regions without preoperative sweating (preoperative HDSS = 1).

| **Segment** | **BTS stability** | **BTS worsening to HDSS 2** | **BTS worsening to HDSS 3‒4** | **UniS stability** | **UniS worsening to HDSS 2** | **UniS worsening to HDSS 3‒4** | **Chi-Square** | **p-value** |
| --- | --- | --- | --- | --- | --- | --- | --- | --- |
| Craniofacial | 38 (90.5) | 4 (9.5) | 0 (0.0) | 47 (60.3) | 21 (26.9) | 10 (12.8) | 12.87 | 0.002 |
| Thoracic | 34 (69.4) | 10 (20.4) | 5 (10.2) | 48 (60.0) | 26 (32.5) | 6 (7.5) | 2.27 | 0.321 |
| Abdominal | 26 (43.3) | 25 (41.7) | 9 (15.0) | 57 (63.3) | 27 (30.0) | 6 (6.7) | 6.52 | 0.038 |
| Hemidorsal | 23 (46.9) | 14 (28.6) | 12 (24.5) | 39 (55.7) | 24 (34.3) | 7 (10.0) | 4.51 | 0.105 |
| Gluteal | 44 (73.3) | 12 (20.0) | 4 (6.7) | 56 (68.3) | 21 (25.6) | 5 (6.1) | 0.61 | 0.736 |
| Inguinal | 40 (76.9) | 12 (23.1) | 0 (0.0) | 57 (67.9) | 21 (25.0) | 6 (7.1) | 4.13 | 0.127 |
| Thighs | 44 (71.0) | 8 (12.9) | 10 (16.1) | 63 (67.7) | 25 (26.9) | 6 (6.5) | 6.86 | 0.032 |
| Feet | 2 (50.0) | 0 (0.0) | 2 (50.0) | 0 (0.0) | 0 (0.0) | 0 (0.0) | NA | NA |
| Hands | 0 (0.0) | 0 (0.0) | 0 (0.0) | 0 (0.0) | 0 (0.0) | 0 (0.0) | NA | NA |
| Axillae | 12 (100.0) | 0 (0.0) | 0 (0.0) | 30 (71.4) | 10 (23.8) | 2 (4.8) | 4.41 | 0.110 |
| Total | 263 (67.4) | 85 (21.8) | 42 (10.8) | 397 (64.0) | 175 (28.2) | 48 (7.7) | 6.73 | 0.035 |

Values are presented as n (%). This table describes new postoperative sweating by anatomical site and surgical strategy. NA indicates that statistical comparison was not applicable because no observations were available in one or both groups.
